# Evaluation of the SpeeDx Carba (beta) multiplex real-time PCR assay for detection of NDM, KPC, OXA-48-like, IMP-4-like and VIM carbapenemase genes

**DOI:** 10.1186/s12879-019-4176-z

**Published:** 2019-07-02

**Authors:** Amanda Bordin, Ella Trembizki, Madeline Windsor, Rachel Wee, Lit Yeen Tan, Cameron Buckley, Melanie Syrmis, Haakon Bergh, Kyra Cottrell, Hosam M. Zowawi, Hanna E. Sidjabat, Patrick N. A. Harris, Graeme R. Nimmo, David L. Paterson, David M. Whiley

**Affiliations:** 1 The University of Queensland, UQ Centre for Clinical Research, Brisbane, Queensland Australia; 2 SpeeDx Pty. Ltd., Brisbane, Queensland Australia; 3 Pathology Queensland Central Laboratory, Brisbane, Queensland Australia; 4 College of Medicine, King Saud bin Abdulaziz University for Health Sciences, Riyadh, Saudi Arabia; 5 World Health Organization Collaborating Centre for Infection Prevention and Control, Riyadh, Saudi Arabia; 6 Gulf Cooperation Council Center for Infection Control, Riyadh, Saudi Arabia; 7 King Abdullah International Medical Research Centre, Riyadh, Saudi Arabia; 8 Griffith University School of Medicine, Brisbane, Queensland Australia

**Keywords:** Multiplex, Real-time, PCR, Carbapenemase, KPC, NDM, OXA-48, IMP-4, VIM

## Abstract

**Background:**

Carbapenemase-producing organisms (CPOs) have emerged as antibiotic-resistant bacteria of global concern. Here we assessed the performance of the Carba (beta) assay, a multiplex real-time PCR assay developed by SpeeDx for the detection of key carbapenemase-encoding genes: KPC, NDM, OXA-48-like, IMP-4-like, and VIM.

**Methods:**

DNA extracts of 180 isolates were tested with the Carba (beta) assay, using previously validated in-house TaqMan probe assays for the relevant carbapenemase genes as the reference standard. The Carba (beta) assay was then directly used to screen 460 DNA extracts of faecal specimens, with positive results subjected to the aforementioned in-house assays plus Sanger sequencing.

**Results:**

The Carba (beta) assay correctly identified the presence of the respective carbapenemase genes in 154 of 156 isolates and provided negative results for all 24 non-CPO isolates. Two isolates provided positive results for OXA-48-like carbapenemase by the Carba (beta) assay only. The Carba (beta) assay had sensitivities of 100% for all targets, and specificities of 100% for KPC, NDM, IMP-4-like, and VIM targets, and 98.5% for OXA-48-like targets. When applied directly to faecal specimens, eight samples were positive by the Carba (beta) assay, two of which were confirmed by in-house TaqMan probe PCR or DNA sequencing.

**Conclusions:**

The Carba (beta) assay is highly sensitive and specific for detecting key carbapenemase genes in isolates. Further testing is required to assess this assay’s suitability for direct screening of clinical specimens.

## Background

Carbapenemase-producing organisms (CPOs) are antibiotic-resistant bacteria of global concern. The United States Centers for Disease Control and Prevention have assigned an “urgent” threat level to carbapenem-resistant Enterobacteriaceae [1], and the World Health Organisation has identified carbapenemase-producing *Klebsiella pneumoniae* and *Escherichia coli* as “bacteria of international concern” [2]. Infections caused by CPOs are, at best, difficult-to-treat, and have sparked concerns over the potential of untreatable infections causing significant morbidity and mortality in health care settings worldwide [2–5]. Rapid detection of CPO infection and subsequent implementation of infection control procedures is pivotal to manage infection and to prevent further nosocomial transmission [6]. Carbapenemase genes of particular clinical importance globally include Klebsiella pneumoniae carbapenemase (KPC), New Delhi metallo-β-lactamase (NDM), oxacillin-hydrolyzing-48-like (OXA-48-like), imipenemase-4-like (IMP-4-like), and Verona integron-encoded metallo-β-lactamase (VIM) genes [3, 5, 7]. In this study, we assessed the performance of the new SpeeDx Carba (beta) multiplex real-time PCR assay for detection of these carbapenemase genes using a panel of CPO isolates. As direct screening of patient samples (particularly rectal swab and faecal samples for intestinal carriage) is beginning to find its way into routine practice to inform infection control [8, 9], we then also applied the method to screen for carbapenemase genes directly in faecal specimens from local patients.

## Methods

### Isolates

The sensitivity and specificity of the Carba (beta) assay for carbapenemase detection was assessed by testing an isolate panel of CPOs (*n* = 156) [10–14] and non-CPOs (*n* = 24) of various bacterial species, and comparing the results to in-house TaqMan probe real-time PCR methods. By origin, CPO isolates were from Pakistan (*n* = 51), Saudi Arabia (*n* = 42), Australia (*n* = 19), Turkey (*n* = 17), USA (n = 17), Bahrain (n = 5) and Qatar (n = 5), and the 24 non-CPO isolates were from Australia. Isolate extracts were prepared using a simple boil-up method whereby single colonies were suspended in 1.0 mL of sterile water to provide a concentration consistent with a 0.5 McFarland standard, before being heated at 95 °C for 30 min.

### Faecal specimens

Remnant DNA extracts from faecal samples (*n* = 460) submitted for routine gastrointestinal testing at Pathology Queensland were tested. In brief, the faecal samples were collected from November 2016 to January 2017 and were extracted at Pathology Queensland using the MagNA Pure 96 System (Roche, Australia) according to manufacturer’s instructions. All samples were tested with the Carba (beta) assay. Samples providing positive results in the Carba (beta) assay were subjected to in-house TaqMan probe PCR assays and Sanger sequencing.

### SpeeDx Carba (beta) PCR

The Carba (beta) assay (SpeeDx Pty Ltd., Australia) was designed to detect KPC, NDM, OXA-48-like, IMP-4-like, and VIM carbapenemase-encoding genes in a single multiplex reaction. The assay utilises PlexZyme (formerly known as MNAzyme) real-time detection technology [15–17] which has advantages in maintaining sensitivity and specificity in multiplex. Detection is predicted for all current known variants of VIM and KPC; NDM-1-7; OXA-48, 162, 163, 181, 204, 232, 244, 245, 247, 370; and IMP-1, 2, 4, 6, 8, 10, 13, 18, 19, 20, 24, 26, 27, 30, 33, 37, 38, 42, 48, 49, 52. The Carba (beta) PCR reaction was prepared as per the manufacturer’s instructions, with 15.0 μL of master mix and 5.0 μL of isolate suspension or faecal extract. Reactions were amplified on the ABI7500 real-time PCR instrument (ThermoFisher Scientific, Australia) using the manufacturer’s cycling conditions: an initial 95 °C 2 min hold, followed by 10 touch-down cycles at 95 °C for 5 s and 61 °C (− 0.5 °C per cycle) for 30 s, followed by 40 cycles at 95 °C for 5 s and 52 °C for 40 s. KPC was reported by FAM, NDM by JOE, OXA-48-like by Texas Red, IMP-4-like and VIM by Cy5 (and therefore IMP-4-like and VIM results could not be distinguished), and the internal control by TAMRA. The internal control DNA was diluted to provide an expected cycle threshold (Ct) value of approximately 22 cycles as per the manufacturer’s instructions.

### In-house PCR

In-house PCR methods utilised previously described primers and probes (see Table 1) for KPC [18], NDM [18], OXA-48-like [19], IMP-4-like [20], VIM [20] and pan-bacterial 16S rRNA [18] (the latter serving as an internal DNA extraction and amplification control). NDM, KPC and 16S rRNA reactions were performed together in a triplex, IMP-4-like and VIM as a duplex, and OXA-48-like as a singleplex reaction. The reverse primer of the OXA-48-like reaction was modified from the original [19] (G to C at position 15) based on sequence data from GenBank. The QuantiFast Probe PCR Kit (QIAGEN, Australia) was used as the basis for the reaction mixes. Each mix comprised 0.5 μM of forward and reverse primers, 0.25 μM of probe and 2.0 μL of isolate preparation or faecal extract in a total reaction volume of 20.0 μL. Reactions were amplified on the Rotor-Gene (QIAGEN, Australia) real-time PCR instrument using an initial 95 °C hold for 3 min followed by 45 cycles at 95 °C for 3 s and 60 °C for 30 s.

**Table 1:** Oligonucleotides for in-house assays in this study

Set	Forward primer (5′-3′)	Reverse primer (5′-3′)	Probe	Reference
KPC^a^	GGCCGCCGTGCAATAC	GCCGCCCAACTCCTTCA	FAM-TGATAACGCCGCCGCCAATTTGT-BHQ1	[18]
NDM^a^	GACCGCCCAGATCCTCAA	CGCGACCGGCAGGTT	VIC-TGGATCAAGCAGGAGAT-MGB-NFQ	[18]
OXA-48-like	GTAGCAAAGGAATGGCAA	CCTTGCTGCTTATTcTCA^c^	FAM-TCC(+A)GA(+G)CA(+C)AA(+C)TACG-Dabcyl	[19]
IMP-4-like^b^	GGCAGTATTTCCTCTCATTT	GCAGCTCATTAGTTAATTCAG	FAM-CATAGTGACAGCACGGGCGGAAT-BHQ1	[20]
VIM^b^	CGCGGAGATTGAGAAGCAAA	AGCCGCCCGAAGGACATC	HEX-TTGGACTTCCTGTAACGCGTGCA-BHQ1	[20]
16S rRNA^a^	TGGAGCATGTGGTTTAATTCGA	TGCGGGACTTAACCCAACA	Quasar670-CACGAGCTGACGACARCCATGCA-BHQ2	[18]

^a^KPC, NDM and 16S rRNA oligonucleotides were tested together in a multiplex PCR as previously described [18]

^b^IMP-4-like and VIM oligonucleotides were tested together in a multiplex PCR as previously described [20]

^c^The lower case base differed to that of the original primer (C instead of G) and was substituted based on OXA-48-like sequence data on the Genbank database

‘+’ indicates locked nucleic acid (LNA) bases; *MGB* minor groove binder, *BHQ* black hole quencher, *NFQ* non-fluorescent quencher

### PCR controls and environmental practices

All PCR test runs included relevant positive control reaction/s to assess PCR amplification and at least two non-template (sterile H_2_O) negative control reactions to check for contamination. Sample extraction, mix preparation, DNA preparation/handling, and PCR amplification were all conducted in separate areas using dedicated equipment.

### Detection limit comparisons

To assess the detection limits of the Carba (beta) assay in comparison to the in-house assays, five CPO isolates were selected as representatives of each carbapenemase gene. DNA extracts of each of these samples were serially diluted ten-fold and run in three to five replicates (see Table 5) through the Carba (beta) assay and the relevant in-house assay. Concentrations were based on the initial isolate extracted at 0.5 McFarland standards (i.e. approximately 1.5 × 10^5^ Colony Forming Units (CFUs) per μL of DNA extract). The detection limit for each target and assay was the lowest dilution where all replicates provided consistent positive Ct values.

### DNA sanger sequencing

Select samples were subjected to DNA sequencing using the primers described in Table 2. Amplification was carried out in 20.0 μL reaction volumes with 0.5 μM of each primer and 2.0 μL of isolate preparation or faecal extract using the QuantiTect SYBR PCR Kit (QIAGEN, Australia) as the basis for the reaction mix. Reactions were cycled on the Rotor-Gene instrument using an initial 95 °C hold at 15 min, followed by 45 cycles of 95 °C for 30 s, 50 °C for 30 s and 72 °C for 60 s, and finally a melt step from 45 to 95 °C at + 0.2 °C/second. PCR products were submitted to the Australian Genomic Research Facility (AGRF; The University of Queensland) for Sanger sequencing and aligned to representative sequences from GenBank.

**Table 2:** Amplification and sequencing primers for Sanger sequencing

Set	Forward primer (5′-3′)	Reverse primer (5′-3′)	Amplicon size (bp)
OXA-48-like	CCTCGATTTGGGCGTGGTTA	TCCGATGTGGGCATATCCAT	475
IMP-4-like	GAAGGTGTTTATGTTCATACTTCGT	TCACTGTGACTTGGAACAACCAGTTTTGC	557
VIM	GAGTTGCTTTTGATTGATACAGC	TCGGTCGAATGCGCAGCACC	291

## Results

### Isolate extracts

The results for isolate extracts are summarised in Table 3. Results from the Carba (beta) assay for 178 of 180 isolates (98.9%) were in concordance with the reference in-house PCR methods. Of these 178 concordant isolates, 154 isolates were correctly-characterised CPOs (16 KPC, 50 NDM, 40 OXA-48-like, 21 VIM, 19 IMP-4-like, six isolates with NDM and OXA-48-like, one isolate with KPC and NDM, and one isolate with OXA-48-like and VIM) and 24 isolates were negative by both the Carba (beta) assay and in-house PCR. A further two isolates provided discrepant results; these two isolates (one *Pseudocitrobacter faecalis* and one *Pseudomonas aeruginosa*) provided concordant results for NDM and IMP-4-like/VIM targets respectively, but were positive for OXA-48-like by the Carba (beta) assay only. Using the in-house assay results as the reference standard, the Carba (beta) assay’s sensitivities and specificities were both 100% for KPC, NDM, IMP-4-like and VIM genes, while for OXA-48-like genes sensitivity was 100% and specificity was 98.5%.

**Table 3:**
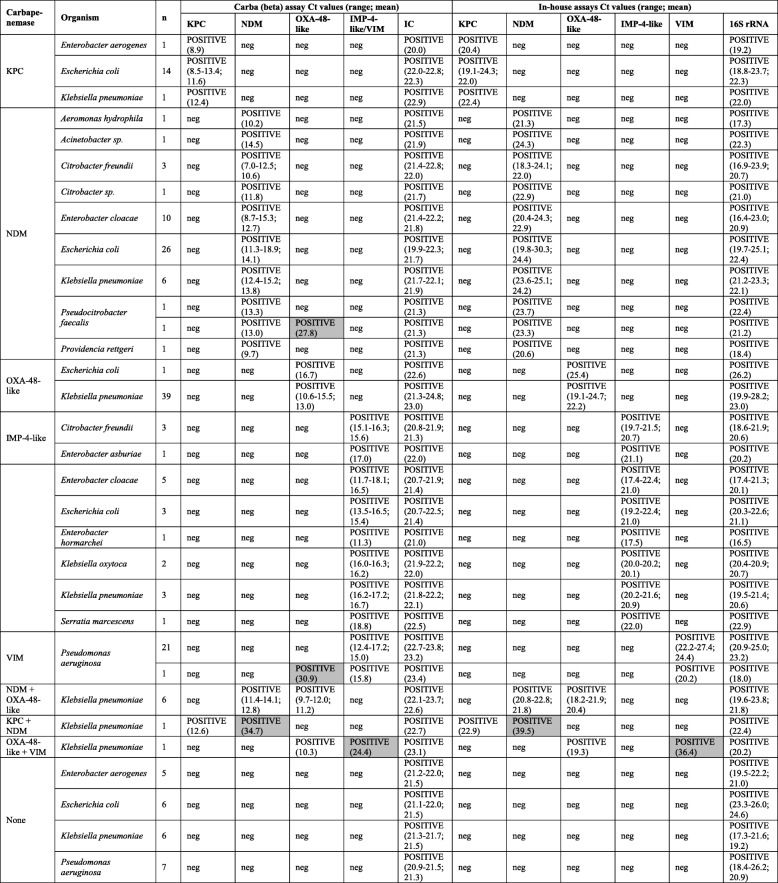
Cycle threshold (Ct) results for isolate extract sample bank (*n* = 180). Results are grouped by carbapenemase type as determined by the Carba (beta) assay, then by species. Results highlighted in grey were negative upon repeated extraction and testing (discussed further in-text)

*neg* negative

An assessment of the Ct values for samples providing concordant positive results (*n* = 154) showed that 152 provided Carba (beta) assay Ct values of less than 20 cycles (7.0–18.9; mean 13.8 cycles). Two isolates (both *K. pneumoniae* and both positive for two genes) provided Carba (beta) assay Ct values of 12.6 cycles (KPC) and 34.7 cycles (NDM) for one isolate and 10.3 cycles (OXA-48-like) and 24.4 cycles (IMP-4-like/VIM) for the second isolate, with in-house PCR assay Ct values of 22.9 cycles (KPC) and 39.5 cycles (NDM), and 19.3 cycles (OXA-48-like) and 36.4 (VIM) respectively. The two isolates providing discrepant results (*P. faecalis* and one *P. aeruginosa*, again both positive for two genes) both provided Ct values < 20 cycles in the Carba (beta) assay for the concordant results (NDM and IMP-4-like/VIM targets respectively), but Ct values of 27.8 or 30.9 cycles for OXA-48-like. These Ct values were the highest observed for OXA-48-like in the Carba (beta) assay.

All four isolates providing Carba (beta) assay Ct values greater than 20 cycles were re-extracted and retested. For each of these isolates, the target originally providing Ct values greater than 20 cycles was negative upon repeat, whereas the results associated with Ct values < 20 cycles were reproducible.

It should be noted that the Carba (beta) assay cycling conditions utilise an initial 10-cycle touchdown step, during which fluorescent signal is not acquired, hence samples tested by Carba (beta) assay typically provided Ct values that were approximately 10 cycles earlier than in-house PCR methods utilising conventional cycling.

### Faecal specimens

Of the 460 faecal DNA extracts tested, 452 were negative for carbapenemase targets by the Carba (beta) assay (see Table 4). Seven samples provided positive results for OXA-48-like (Ct values 25.1 to 28.9 cycles), and one sample for IMP-4-like/VIM (25.4 cycles). When tested by the in-house assays, the seven OXA-48-like samples provided negative results, whereas the IMP-4-like/VIM positive sample produced a positive result in the IMP-4-like PCR (Ct value of 31.4 cycles). DNA sequencing was attempted for all eight samples; one of the OXA-48-like positive samples returned a sequence confirmed as OXA-181, and the IMP-4-like/VIM positive sample returned an IMP-4 sequence. Amplification for DNA sequencing was not successful for the other samples.

**Table 4:** Results for faecal swab clinical extracts (*n* = 460). Regarding ‘n/a’ results, only clinical extracts positive by the Carba (beta) assay were subjected to in-house assays and DNA sequencing

n	Carba (beta) assay Ct values (range; mean)					In-house assays Ct values (range; mean)						Sequencing result
	KPC	NDM	OXA-48-like	IMP-4-like/VIM	IC	KPC	NDM	OXA-48-like	IMP-4-like	VIM	16S rRNA	
1	neg	neg	neg	POSITIVE (25.4)	POSITIVE (22.6)	n/a	n/a	n/a	POSITIVE (31.4)	neg	POSITIVE (11.2)	IMP-4
1	neg	neg	POSITIVE (27.0)	neg	POSITIVE (23.4)	n/a	n/a	neg	n/a	n/a	POSITIVE (15.5)	OXA-181
6	neg	neg	POSITIVE (25.1–28.9; 27.1)	neg	POSITIVE (22.3–24.4; 23.0)	n/a	n/a	neg	n/a	n/a	POSITIVE (12.4–18.7; 14.2)	n/a
452	neg	neg	neg	neg	POSITIVE (21.4–28.7; 22.8)	n/a	n/a	n/a	n/a	n/a	n/a	n/a

*neg* negative

*n/a* not available

### Assay detection limits

The Carba (beta) assay detection limit was the same for NDM, KPC and VIM targets, and was more sensitive for OXA-48-like and IMP-4-like targets, in comparison to the corresponding in-house assays (see Table 5). The detection limits for the Carba (beta) assay were approximately 1.5 CFU/μL for all five targets. Detection limits for the in-house assays were approximately 15 CFU/μL for IMP-4-like and OXA-48-like assays and 1.5 CFU/μL for KPC, NDM and VIM assays.

**Table 5:**
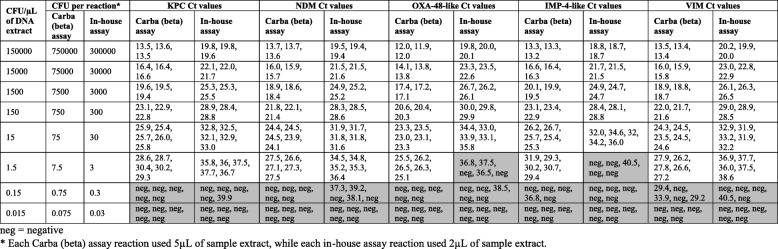
Detection limits for Carba (beta) assay and in-house assays. Performance was assessed individually (i.e. with five isolates possessing only one of the assayed genes) using isolate extracts characterised in this study. All Ct values for replicates at each dilution level are shown. Results beyond the limit of detection for each assay and gene are shaded in grey

*neg* negative

*Each Carba (beta) assay reaction used 5 μL of sample extract, while each in-house assay reaction used 2 μL of sample extract

## Discussion

The results indicate the Carba (beta) assay is suitable for detection of carbapenemase genes in cultured isolates. Correct results were provided for 154 of 156 CPO isolates and 24 of 24 non-CPO isolates, giving the Carba (beta) assay 100% sensitivity and ≥ 98.5% specificity for cultured isolate extracts. From a practical standpoint, the single reaction format of the Carba (beta) assay offers significant advantages in terms of isolate testing throughput over our current in-house methods, which currently require three reactions per isolate.

The reduced OXA-48-like specificity observed for the Carba (beta) assay was due to two isolates (*P. faecalis* and *P. aeruginosa*) that gave OXA-48-like Ct values of 27.8 and 30.9 cycles. These were negative by the OXA-48-like in-house assay and DNA sequencing, and subsequently provided negative results in the Carba (beta) assay when re-extracted and retested. Negative results following re-extraction and retesting were also observed for a further two isolates that initially provided Ct values of 34.7 cycles and 24.4 cycles for NDM and VIM respectively in the Carba (beta) assay. Such high Ct values are indicative of low DNA loads, and likely suggests these isolates were contaminated with other genomic DNA during the original sample preparation. Employing a Ct value cut-off in the Carba (beta) assay may help to address these issues, but additional experimentation would be needed to establish appropriate cut-offs for the isolates and different types of clinical samples.

The initial results from the direct faecal sample testing suggest the Carba (beta) assay may be suitable for screening purposes, but more work is needed here. The majority of samples (98.3%; 452/460) returned negative results. This low detection rate is consistent with previous studies indicating these genes are at low prevalence in our local Australian population [21]. However, of the eight positive samples by the Carba (beta) assay (seven for OXA-48-like and one for IMP-4-like/VIM), only two of these positives were confirmed by in-house PCR and/or DNA sequencing (one IMP-4 and one OXA-181). It is possible that OXA-48-like genes may be present in the six non-confirmed samples, and were only detected by the Carba (beta) assay due to its more sensitive detection limit; as seen in Tables 4 and 5, the OXA-48-like Ct values provided by the faecal samples were similar to the Ct value for the limit of OXA-48-like detection. Alternatively, these may be due to contamination as discussed earlier. We were unable to investigate this further as, unlike the isolates, the original faecal samples were not available for re-extraction and retesting. Nevertheless, further validation of direct sample testing is warranted, including from patients at higher risk of harbouring CPOs. In this context, a limitation of this investigation was that we only tested a bank of faecal samples from patients with suspected gastrointestinal infection from a low-CPO prevalence setting. An additional limitation of this study was that we did not assess the detection limit of the assays for each of the individual gene variants.

## Conclusion

In summary, the Carba (beta) assay developed by SpeeDx was sensitive and specific for detecting carbapenemase genes in isolates. Applying a Ct value cut-off when testing isolate extracts may be useful to enhance performance. Further studies are required to determine the suitability of this assay for direct testing of clinical samples.

## Data Availability

The datasets for this study are available from the corresponding author on reasonable request. Materials for this study are retained at the University of Queensland Centre for Clinical Research, Australia.

## Abbreviations

AGRF: Australian Genomic Research Facility
CFU: Colony Forming Unit
CPO: carbapenemase-producing organism
Ct: Cycle threshold
IC: internal control|
IMP: imipenemase
KPC: *Klebsiella pneumoniae* carbapenemase
NDM: New Delhi metallo-β-lactamase
OXA: oxacillin-hydrolyzing
rRNA: ribosomal ribonucleic acid
VIM: Verona integron-encoded metallo-β-lactamase
